# Heat-Induced Preparation of Myofibrillar Protein Gels Reinforced Through Ferulic Acid, α-Cyclodextrin and Fe(III)

**DOI:** 10.3390/foods14081290

**Published:** 2025-04-08

**Authors:** Ziyi You, Yushan Chen, Wendi Teng, Ying Wang, Yuemei Zhang, Jinxuan Cao, Jinpeng Wang

**Affiliations:** 1 Key Laboratory of Geriatric Nutrition and Health, Beijing Technology and Business University, Ministry of Education, Beijing 100048, China; 15564060687@163.com (Z.Y.); 19811715330@163.com (Y.C.); wenditeng@btbu.edu.cn (W.T.); wang-ying@btbu.edu.cn (Y.W.); zhangyuemei@btbu.edu.cn (Y.Z.); caojinxuan@btbu.edu.cn (J.C.); 2 School of Food and Health, Beijing Technology and Business University, Beijing 100048, China; 3 College of Food and Biological Engineering, Chengdu University, Chengdu 610106, China

**Keywords:** ferulic acid, metal–phenolic acid, myofibrillar protein hydrogel, cross-linking network structure

## Abstract

Phenolic acids have a positive effect on the processing quality of myofibrillar protein (MP) gels. However, in this study, the addition of ferulic acid (FA) did not have a positive effect on MP gels. To address this issue, we performed the addition and observed the effects on the structure of MP gels by both surface coating and internal cross-linking: addition of FA alone, addition of α-cyclodextrin (CD) to encapsulate FA (MP-FA/CD), and addition of Fe(III) to form a metal–phenolic network structure (Fe @MP-FA) and a metal–cyclodextrin–phenolic acid structure (Fe@MP-FA /CD). It was found that both Fe @MP-FA formed by surface coating and internal cross-linking were able to improve the textural properties of MP gels, including hardness, elasticity, chewability, adhesion, etc. FA effectively promoted the conversion of some of the non-fluidizable water to the bound water morphology, and the addition of Fe(III) effectively enhanced this trend. In particular, the composite network structure formed by Fe@MP-FA/CD more significantly promoted the conversion to bound water and improved the water retention of the gel. Hydrophobic interactions and hydrogen bonding in non-covalent bonding as well as disulfide bonding in covalent bonding were always the main factors promoting the formation of gels from MP after different additions. Meanwhile, different gel treatments lead to changes in the structure of different proteins. Internal cross-linking with the addition of FA promotes protein oxidation, whereas CD reduces the occurrence of oxidation and promotes a homogeneous gel structure. Surface coating with the addition of FA/CD resulted in a reduction in pores in the MP gels and a denser gel structure. However, the addition of internal cross-linking resulted in a gel with a loose and rough network structure. In this study, we compared the common methods of gel enhancement, with the objective of providing a reference for the improvement in the gel texture of meat products.

## 1. Introduction

Myofibrillar proteins are essential for the formation of a three-dimensional gel network, which has a crucial role in the texture of meat products, such as chewability, elasticity and slicing ability [1,2]. Protein molecules are denatured and unfolded after heating, exposing more relevant groups, which are then aggregated through hydrophobic interactions to form a three-dimensional reticulated protein gel structure. Thus, they support the structure of the meat product and slow down the loss of water from the tissue. Myofibrillar proteins include mainly actin and myosin. These two form myonodes by forming in an overlapping manner, which then form myocytes and further form myogenic fibers. Gel formation occurs primarily through myosin, which is extremely unstable and easily oxidized. In addition to the oxidation that normally occurs, meat products are enriched with lipolytic compounds that contribute to the oxidation process, causing adverse effects on the meat product through the production of reactive oxygen species that lead to protein aggregation and amino acid modification. However, meat products are prone to oxidation during processing, which can lead to alterations in protein structure. This is primarily due to modifications in amino acid side chains, including the formation of protein carbonylation, protein fragmentation, and cross-linking [3]. This disruption can compromise the integrity of the gel network, leading to disarray, surface irregularities, diminished color vibrancy, and reduced water retention [4,5], further reducing the quality of the meat [6]. It can even trigger oxidative stress within the body following consumption [7], so resistance to protein oxidation has a very important role.

The addition of polyphenols and polysaccharides can effectively inhibit the oxidation of myofibrillar proteins and enhance their gel properties. Polyphenols are regarded as a safe food additive that inhibits oxidation reactions by lowering oxygen levels, neutralizing reactive oxygen species (ROS), and chelating metal ions. [8,9]. Polyphenols can have covalent and non-covalent interactions with proteins. Covalent interactions generally occur under oxidizing conditions, where polyphenols are oxidized to semiquinones or quinones. The polyphenols are then reacted with protein side-chain groups to cross-link to form complexes. Under non-oxidizing conditions, non-covalent cross-links are formed mainly through hydrophobic interactions and hydrogen bond formation. Ferulic acid, a type of plant phenolic acid, markedly diminishes oxidation-induced alterations in MPs and boosts the cross-linking of myofibrillar proteins [10]. However, the application of FA is constrained by its low solubility in water, inefficient utilization, and susceptibility to instability. Complexation of FA with CDs can solve the problem [11]. It also has a large concentration dependence, with high concentrations adversely affecting meat products. Cyclodextrin-embedded polyphenols were able to inhibit the oligomerization of proteins while improving the stability of polyphenols. Cyclodextrins possess an inner hydrophobic cavity and an outer hydrophilic surface, enabling them to form noncovalent inclusion complexes with a range of guest molecules. The interaction between the host cyclodextrin and the guest molecules occurs via forces such as van der Waals interactions, hydrophobic effects, and hydrogen bonding, which serve to enhance the solubility and molecular stability of the guest molecules [12]. Polysaccharides and polyphenols have great affinity for each other and can form complexes through hydrophobic interactions and hydrogen bonding. At the same time, the addition of polysaccharides in gel formation can play the role of water retention and filling, and the gel network formed is more dense.

However, polyphenols are concentration-dependent in gels; when the concentration is too high, it will modify proteins excessively and have a negative effect on the gel [13]. The addition of metal ions can effectively improve the chemical shifts of protein amino acid residues and the functional properties of protein molecules. It also forms a metal–phenol network structure with polyphenols and improves the properties of the gel. Metal ions prompt proteins to aggregate into larger complexes, leading to a denser and more uniform structure of the gel [14]. The addition of CaCl_2_ enhances the cross-linking of surimi myofibrillar proteins, thereby densifying the structure [15]. The addition of alkali denatured and aggregated egg white proteins, followed by the introduction of low concentrations of Ca^2+^, Zn^2+^, and Fe^3+^, enhance the gel’s strength and structural ordering [16]. In the mixed system of pea protein and cod protein, the addition of NaCl and CaCl_2_ diminishes the negative charge on the proteins, thereby reducing repulsive forces. This results in the formation of larger aggregates and gels that exhibit enhanced elasticity and adhesion [17].

The oxidation of meat products leading to deterioration in their gel texture can greatly affect product quality. Although the antioxidant effect of FA on myofibrillar proteins has been reported, the improvement in gel texture varies greatly depending on the concentration and other factors. In contrast, CD is able to encapsulate polyphenols and eliminate the negative effect of excessive cross-linking on the protein in the gel texture. Therefore, it was hypothesized that FA/CD had the same improvement effect on MP gels. Meanwhile, the metal ion Fe(III) was able to form a metallophenolic network structure with polyphenols. Similarly, it is hypothesized that Fe(III) is equally capable of promoting the improvement in MP gel structure. Existing studies mainly discuss this between other individual proteins, but a few discuss the relationship between FA, CD, Fe(III) and MP gel structure and the effect of different composite structures on the gel structure. So the aim of this study was to explore the effects of FA, the addition of CD to FA for encapsulation (MP-FA/CD), and the addition of Fe(III) to form a metal–phenolic network structure (Fe@MP-FA) and metal–cyclodextrin–phenolic acid structure (Fe@MP-FA/CD) on the structure of MP gels. The effect of different coating methods after treating the gels was also considered. Thus, it provides a reference for the improvement of MP gel texture in meat products.

## 2. Materials and Methods

### 2.1. Materials and Chemicals

Fresh chicken breasts were purchased from local supermarkets, stored at 4 °C and myofibrillar proteins were extracted within 24 h. NaCl, MgCl_2_, NaH_2_PO_4_, Na_2_HPO_4_, NaOH, EGTA, ferulic acid, α-cyclodextrin, FeCl_3_−6H_2_O, anhydrous ethanol, urea, β-mercaptoethanol, Caumas Brilliant Blue, and other chemicals (analytical grade) were purchased from Aladdin Biochemistry and Technology Co. Ltd. (Shanghai, China).

### 2.2. Preparation of Myofibrillar Protein Gel

#### 2.2.1. Extraction of Myofibrillar Protein

The fat and connective tissue were removed from the chicken breasts and placed in a meat grinder. After the meat was minced, it was combined with 4 times the volume (*v*/*w*) of separation buffer (0.1 M NaCl, 2 mM MgCl_2_, 1 mM EGTA, 10 mM NaH_2_PO_4_/Na_2_HPO_4,_ pH 7.0), homogenized and centrifuged at 10,000 *g* for 15 min at 4 °C. Then, the precipitate was collected, the above operation was repeated twice and the resulting precipitate was MP [18]. The protein concentration was determined by BCA method. The extracted MP was stored at 0~4 °C and used up within 48 h.

#### 2.2.2. Ferulic Acid (FA)/Cyclodextrin (CD) Inclusion Complex Preparation

The FA ethanol solution was added dropwise to 2 mM CD solution (molar ratio of CD:FA: 1:1) at 60 °C and 400 rpm and stirred continuously for 2 h, sealed and light protected. Then, it was dried at 60 °C and washed with anhydrous ether 3 times to remove the residual FA. The powder was then dried naturally at room temperature, sealed in a plastic bag and stored in a desiccator.

#### 2.2.3. Preparation of Myofibrillar Protein (MP)-Ferulic Acid (FA) Composite Gel

MP was prepared to a protein concentration of 40 mg/mL using 0.6 M NaCl. The FA and its inclusion complex were subsequently dissolved in the MP solution. This resulted in the final concentration of FA and its inclusion complex reaching 0.04% (*w*/*v*). A control group was established using MP solution without the addition of FA and its inclusion complex. Following this, the MP solution underwent heating via a secondary method. The samples were initially placed in a water bath maintained at 30 °C for 20 min. Subsequently, the temperature was increased linearly from 30 °C to 70 °C at a rate of 1 °C per minute, and the samples were then held at 70 °C for an additional 20 min. Finally, the gel was promptly cooled in ice water and stored in a refrigerator at 4 °C overnight [19].

#### 2.2.4. Preparation of Fe(III)-Reinforced Myofibrillar Protein (MP) Gels

Surface coating: the surface construction method of MPN used is referred to in [20]. Initially, the MP gels were prepared and subsequently submerged in deionized water. Following this, equivalent volumes of FA (4 mg/mL) and FeCl_3_−6H_2_O (1.2 mg/mL) solutions were sequentially introduced into the system, achieving final concentrations of FA at 0.4 mg/mL and FeCl_3_−6H_2_O at 0.12 mg/mL. After each addition, the mixture was agitated for 30 s to ensure thorough contact between the FA and FeCl_3_−6H_2_O solutions and the gel samples, after which the pH was adjusted to 10.0 using NaOH (2 M). Finally, the coating procedure was performed five times, with the gels being rinsed multiple times with deionized water throughout the process. The final gel samples obtained were Fe@MP-FA and Fe@MP-FA/CD.

Internal cross-linking: FA (4 mg/mL) and FeCl_3_−6H_2_O (1.2 mg/mL) solutions were added to the MP solution and mixed homogeneously, making the final concentrations of FA and FeCl_3_−6H_2_O up to 0.4 mg/mL and 0.12 mg/mL. Then, the pH was adjusted to 10.0 with NaOH (2 M). The secondary heating method was used to prepare the gels using the same steps.

### 2.3. Textural Properties

The MP gel was measured by *p*/20 probe at room temperature and the parameters were as follows: using the texture profile analysis (TPA) mode, pre-test speed: 2 mm/s, test speed: 0.8 mm/s, post-test speed: 0.8 mm/s, trigger force: 5.0 g, interval time: 5.0 s, target deformation: 50%.

### 2.4. Cooking Yield and WHC

First, the MP sol was heated in the beaker with cling film during heating to prevent the surface of the MP gel from drying out. W is the weight of the empty beaker, W1 is the total weight of the beaker and MP sol before the heat-induced treatment. and W2 is the total mass of the beaker and MP gel after the heat-induced treatment. Cooking yield is calculated using the following [21].

Cooking yield (%) = (W_1_ − W_2_)/(W_1_ − W) × 100% (1)

The gel samples were centrifuged at 10,000 *g* for 10 min at 4 °C. The M_0_ is the weight of empty centrifuge tube, M_1_ is the total weight of centrifuge tube and MP gel before centrifugation, and M_2_ is the total weight of tube and MP gel after centrifugation. WHC is calculated using the following equation [22]:

WHC (%) = (M_2_ − M_0_)/(M_1_ − M_0_) × 100% (2)

### 2.5. Water Distribution

Water composition and distribution of MP gel samples were analyzed using LF-NMR [23]. First, 2 g of MP gel was put in a glass tube. The spectrum was collected using an LF-NMR analyzer with 21 MHz of resonance frequency at 32 °C. The transverse relaxation time (T_2_) was measured using a Carr–Purcell–Meiboom–Gill (CPMG) with 12,000 echoes, 32 scans, 6.5 s between scans, the lengths of the pulse set at 200 μs. Each test was repeated 4 times. The distribution of T2 and the percentage of peak area were calculated for each sample.

### 2.6. Dynamic Rheological Characterization

Initially, the MP solution was uniformly applied to the center of the carrier table, employing an oscillating temperature scanning test mode with a P20-type rotor for analysis. The parameters were set as follows: the oscillation frequency was 0.1 Hz, the maximum stress was 2%, the gap between the upper and lower plates was 1 mm, and the sample was heated at a rate of 1 °C/min. Finally, the heating curves of the samples from 20 to 80 °C were recorded, along with the values of the energy storage modulus (G’), loss modulus (G”), and phase angle tangent (tanδ).

### 2.7. Covalent and Non-Covalent Interactions

First, 1 g of gel sample was added to solutions, then the following reagents were added separately: 9 mL of 0.05 M NaCl (SA), 0.6 M NaCl (SB), 0.6 M NaCl + 1.5 M urea (SC), 0.6 M NaCl + 8 M urea (SD), 0.6 M NaCl + 8 M urea + 2% β-mercaptoethanol (SE) mixing. After homogenization for 20 s, the samples were centrifuged at 8000× *g* for 10 min at 4 °C. Then, the supernatant was taken to determine the protein content by Bradford’s method. The non-specific binding between protein molecules was indicated by the protein content in SA solution. Ionic bonding, hydrogen bonding, hydrophobic interactions, and disulfide bonding were expressed by the difference in protein content between SB and SA solution, SC and SB solution, SD and SC, and SE and SD. Results are expressed as grams of soluble protein per liter of homogenate [24].

### 2.8. Secondary Structure

Secondary structure changes in MP samples were analyzed using MOS-500 circular dichroism spectrometer [25]. MP gels were dissolved in 0.6 M NaCl phosphate buffer (pH 7.0) and centrifuged at 8000× *g* for 10 min. Then, the supernatant was removed and the protein concentration was adjusted to 0.2 mg/mL, adding the samples to the 1 mm quartz cuvette (scanning temperature: 25 °C, scanning wavelength: 190~260 nm, scanning rate: 100 nm/min, bandwidth: 1 nm).

### 2.9. Microstructure

The MP gels were fixed with liquid nitrogen for 24 h, then were freeze-dried and sprayed with gold. The microscope was accelerated at a voltage of 5 kV to observe [26].

### 2.10. Elemental Analysis

The distribution of Fe(III) on the surface of MP gels was examined using the SEM-EDS. The MP gels were subjected to liquid nitrogen freezing treatment and then freeze-dried in vacuum using a freeze-dryer. The gels were fixed on the sample stage by conductive adhesive and sprayed with gold coating, and then observed and photographed under an operating voltage of 5 kV.

### 2.11. Statistical Analysis

All measurements were performed three times and expressed as means ± SD. Statistical analysis was performed using SPSS 19.0 (SPSS, Inc., Chicago, IL, USA) software. Significant differences between data means (*p* < 0.05) were tested using Duncan (D); Graphs were plotted using Origin 2021 (OriginLab, Co., Ltd., Northampton, MA, USA).

## 3. Results and Discussion

### 3.1. The Impact of Ferulic Acid (FA) and Iron(III) on Gel Properties

#### 3.1.1. Textural Property

The application of FA significantly influenced the gel hardness (*p* < 0.05), varying with the treatment applied. It was demonstrated that the application of a surface coating enhanced its properties, whereas internal cross-linking diminished them (Table 1 and Table 2). This may be due to different levels of protein aggregation [27]. The gel’s elasticity, chewability, adhesion, and recovery displayed a comparable trend to its hardness, while the gel’s cohesion remained relatively stable and did not experience significant alterations (*p* > 0.05). The addition of both treatments, FA and CD, had minimal impact on the textural properties. (*p* > 0.05). The presence of Fe(III) influenced the gel texture to varying degrees, contingent upon the treatment applied. In the surface-coated group, no substantial alterations were observed (*p* > 0.05). Conversely, within the internally crosslinked group, there was a marked enhancement in hardness, adhesion, mastication, gelation, and recovery, all of which were statistically significant (*p* < 0.05).

In summary, the application of FA through surface coating proved to be more effective in enhancing the textural properties of the gels compared to internal cross-linking. Furthermore, Fe(III) played an enhanced role, which is consistent with the findings of Zhao et al. [28]. It is possible that Fe(III) may enhance the interaction between FA and MP, leading to the formation of a denser three-dimensional network structure [29], and restrict the movement of the bound water within the gel matrix [17]. Therefore, it possesses the capability to withstand external pressures and the ability to revert to its original state. Research has demonstrated that sufficient binding between polyphenols and proteins can augment the structural integrity of protein gels. For instance, Sun et al. discovered that apple polyphenols can improve the gel properties of grass carp surimi. This phenomenon is attributed to the hydrophilic nature of the hydroxyl groups present within the polyphenols, leading to enhanced hydration.

#### 3.1.2. Cooking Loss and WHC

The cooking loss rate serves as an indicator of moisture loss in meat when subjected to heat. Given that the surface coating treatment was executed after the completion of heat-induced gel preparation, the cooking loss for this particular group was undeterminable (Table 3) and, therefore, is not addressed in the discussion. The cooking losses for both FA and FA/CD increased (Table 4); however, neither increase was statistically significant (*p* > 0.05). This suggests that the presence of FA at a specific concentration exerts a minimal impact on the MP structure and does not lead to an increase in water loss. The reduced cooking loss observed in the gel with Fe(III) compared to the control group could be attributed to the interaction between Fe(III) and ferulic acid (FA), which leads to the formation of a metal–phenolic network [30], which offers superior thermal stability and adsorption capabilities, while also reducing the cooking loss of the gel [31].

Water-holding capacity (WHC) refers to the ability of meat to retain water, a factor that is intrinsically linked to the texture and color of meat products. When subjected to heat and subsequently cooled, the majority of water becomes entrapped within the three-dimensional, dense network structure created by myofibrillar proteins (MP). The greater the WHC value, the less likely it is for water to be expelled from the gel. The addition of FA and FA/CD affects the gels’ WHC differently depending on the treatment compared to the control. The application of a surface coating resulted in a substantial enhancement (refer to Table 3) (*p* < 0.05), whereas internal cross-linking exhibited no significant alteration (refer to Table 4) (*p* > 0.05). Additionally, the presence of Fe(III) did not lead to a significant change in water-holding capacity (WHC) when compared to the group that received no such addition (*p* > 0.05). This phenomenon could be attributed to the oxidation of protein gels, wherein hydroxyl radicals trigger the unfolding of the α-helical structure, resulting in hydrogen bonding. Consequently, this leads to the formation of MP gels with diminished water-holding capacity (WHC) following oxidation [8,32]. FA possesses a robust capacity to sequester free radicals, thereby mitigating the oxidative denaturation of proteins to a significant degree [33]. Furthermore, FA can either extend or shield the protein structure, enhance the cross-linking capability of proteins, and decelerate the alteration in the MP structure [34], and improve the water retention of the gel. The enhancement in the gels’ water-holding capacity (WHC) through the use of FA/CD could be attributed to α-CD shielding the MPs against oxidative damage and enhancing their solubility [35], the reduction in excessive cross-linking of proteins, thereby increasing water retention within the gel [36].

#### 3.1.3. Water Distribution

**Figure 1:**
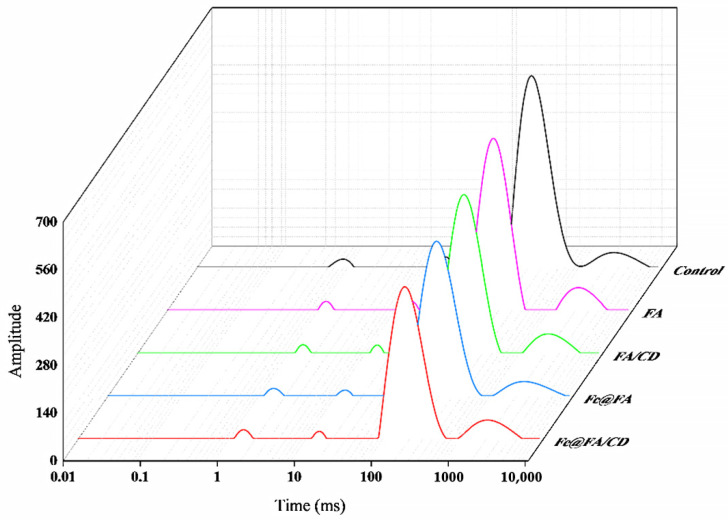
The distribution of T_2_ relaxation time in MP gel with FA or FA/CD addition and Fe(III) strengthening (internal crosslinking).

NMR is capable of analyzing the water state within MP gel systems. [37]. Relaxation time is the time required for food to regain its equilibrium state after it has been shifted by a transient external disturbance. The shorter the relaxation time, the more stable and less mobile the state of moisture. T_2_ reflects the water distribution status within the MP gel. A shorter relaxation time signifies a more rapid exchange process and indicates stronger interactions [38]. As illustrated in Figure 1, four distinct peaks emerge within the gel across the T_2_ range of 0.01 to 10,000 milliseconds, with each peak corresponding to a unique water state. These four components: T_2b_ (0.1–1 ms, bound water that is tightly bound to the protein molecules), T_21_ (1–10 ms, partially immobile water), T_22_ (10–1000 ms, immobile water), and T_23_ (1000–10,000 ms, free water). The corresponding peaks at T_2_ are noted as P_2b_, P_21_, P_22_, and P_23_, respectively.

The water molecule relaxation times of the different gels after addition in both surface coating and internal crosslinking are shown in Table 5 and Table 6. Both ways of adding FA/CD and additionally adding Fe(III) effectively prolonged the relaxation time of T_2B_ and decreased the relaxation time of T_21_. This indicates that the original effect related to bound water in the gel is weakened. Bound water mainly acts through hydrogen bonding and with myofibrillar proteins and phenolic groups in FA as hydrogen donors can also form hydrogen bonds with C=O groups in proteins [39]. So it is hypothesized that there may be a competitive interaction weakening the interaction of the MP protein with water molecules, as well as FA covering the protein surface and inhibiting the interaction with water molecules [40]. The metal ion Fe(III) may have interfered with the hydrogen bonding between MP and water molecules, leading to the weakening of the interaction between MP and water molecules [41]. CD-embedded FA mainly plays a role in promoting the protection of MP from oxidative attack and increasing its solubility, while ligand bonding with metal ions may also interfere with the presence of the original water molecules [42].The decrease in the relaxation time of T_21_, T_22_, and T_23_ indicates the enhanced interaction of the gel with some of the non-flowing water, non-mobile water, and free water. In particular, T_21_, T_22_, and T_23_ decreased significantly with the addition of Fe(III). This indicates that the presence of Fe(III) effectively enhanced the interaction between this part of the water molecules and the gel network. This may be enhanced by attraction with polar groups in myofibrillar proteins through electrostatic interactions. Also, the formation of a denser gel network structure may have facilitated the retention of water molecules.

Table 7 and Table 8 shows the ratio of water molecule relaxation peaks of different gels after addition by both surface coating and internal cross-linking. It was found that the addition of FA and FA/CD could effectively increase the content of bound water on the basis of MP gels in two ways, but the cyclodextrin encapsulation had little effect. Therefore, the addition of FA is able to promote the transition of more water to the bound state. This is because FA adducts with protein side-chain groups to form protein–polyphenol aggregates, thereby promoting the cross-linking of proteins to form a denser gel network. This also enhances the adsorption of polar groups and increases the proportion of bound water [43]. The addition of Fe(III) was able to effectively increase the bound water content again. It is hypothesized that it is because Fe(III) forms a supramolecular network structure with ferulic acid through coordination bonds to further enhance the gel network. In the presence of Fe(III), cyclodextrin plays a facilitating role. It is possible that the metal-ligand interaction between Fe(III) and CD molecules can stabilize the CD, thus promoting the gel formation. The system with the addition of Fe(III) can effectively promote the transition to bound water. It suggests that the polyphenol and metal ion systems play a more important role.

The low-field nuclear magnetic resonance (LF-NMR) results showed that FA could effectively promote the conversion of water to bound water, and the addition of Fe(III) effectively enhanced this trend. In particular, the composite network structure formed by the addition of CD after the formation of the metal phenolic network by Fe(III) and FA was the most effective in promoting the transition to bound water. This enhances the interaction between MP gels and water molecules, promotes the formation of a denser gel network structure, and allows MP gels to retain more water

#### 3.1.4. Dynamic Rheological Alterations

**Figure 2:**
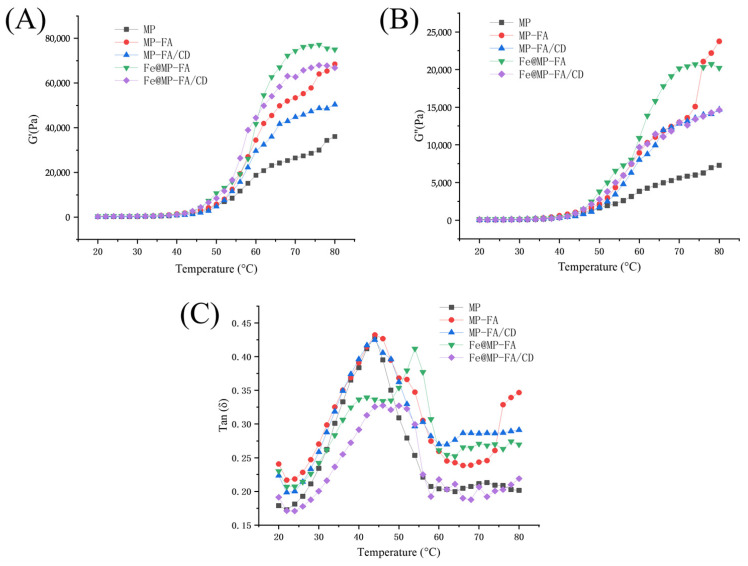
The rheological properties of MP gel with different treatments: (**A**) storage modulus G’; (**B**) loss modulus G”; (**C**) tan (δ) value.

The dynamic rheological alterations in myofibrillar proteins subsequent to the introduction of ferulic acid and iron ions were quantified, as depicted in Figure 2. Both (A) and (B) illustrate that the trends of G’ and G” are congruent. When the heating temperature remains below 40 °C, both G’ (storage modulus) and G” (loss modulus) remain constant. This phase represents the initial stage of gel formation, characterized by the movement and aggregation of myosin following heating. Given the weak movement, it is insufficient to induce changes in G’ and G”. Between 40 °C and 50 °C, G’ and G” exhibit a gradual increase. Upon exceeding 58 °C, both G’ and G” exhibited a pronounced upward trend. At temperatures above 50 °C, myosin begins to unfold and interact, forming a gel network structure. When the heating temperature passes 58 °C, the protein undergoes heat denaturation. This promotes hydrophobic interactions and disulfide bond formation, leading to a rapid increase in G’ and G”. A stable three-dimensional protein network structure was initially formed at 80 °C [44]. The G” value consistently remained lower than the G’ value throughout the heating process, suggesting that the protein gel system exhibits greater elasticity compared to viscosity. However, it is important to note that the two properties are positively correlated.

Upon the addition of FA and FA/CD, both the storage modulus (G’) and loss modulus (G”) exhibited increases compared to the control group. Following reinforcement with Fe(III), the values of G’ and G” peaked within the FA group. This phenomenon occurs because FA induces unfolding of the MP structure, which increases the degree of cross-linking, resulting in a denser MP gel. Consequently, the resultant gel is more compact and stable, leading to heightened viscoelastic properties.

The phase angle tanδ serves as an indicator of rheological alterations. It represents the ratio of the loss modulus (G”) to the storage modulus (G’). A higher ratio indicates greater viscosity. It can reflect the viscoelastic properties of the gel [45]. As illustrated in Figure 2C, the tanδ values for all samples are below 1. This signifies that the storage modulus (G’) of the samples passes the loss modulus (G”), suggesting that the gel exhibits more elasticity than viscosity. Furthermore, tanδ exhibits a trend of initial increase followed by a subsequent decrease as the temperature rises, aligning with the transformation mechanism of MP [46]. For the MP samples, tanδ increased significantly between 30 and 45 °C. The results showed that the viscosity of the gels was higher than the viscosity at the initial heating stage. This indicates the transformation of viscous proteolysis into elastic semi-solid during the initial heating stage. After peaking at about 45 °C, tanδ decreased rapidly, indicating myosin change and gel formation. The addition of FA and FA/CD significantly increased tanδ, indicating that FA improved the elasticity of MP gel.

These results showed that the rheological properties of myofibrillar proteins were improved due to FA [47].

### 3.2. Effect of Ferulic Acid and Fe(III) on Gel Structure

#### 3.2.1. Covalent and Non-Covalent Interactions

**Figure 3:**
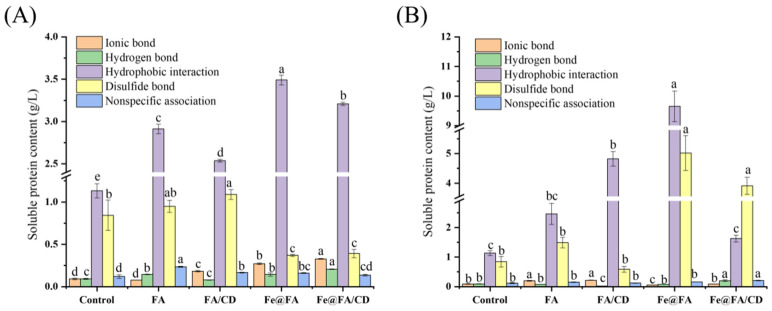
Effects of FA or FA/CD addition and Fe(III) strengthening on chemical forces of MP gel: (**A**) internal crosslinking; (**B**) surface coating. Different letters indicate significant differences between groups (*p* < 0.05).

As shown in Figure 3, the hydrophobic interaction was the strongest among the effects in maintaining the gel structure, followed by disulfide bonding, and the contribution of both was significantly greater than that of ionic and hydrogen bonding (*p* < 0.05). This indicates that hydrophobic interactions and disulfide bonds play a major role in the formation of MP gels [48]. This contributes to the formation of protein aggregates, which improve the homogeneity and densification of the gel network [49]. The addition of FA/CD by internal cross-linking as well as surface coating with Fe@FA and Fe@FA/CD significantly increased the disulfide bond content compared to the blank control group. It may be due to the encapsulation of cyclodextrins blocking the interaction between FA and proteins [50]. This led to a decrease in antioxidant capacity. This led to the oxidation of sulfhydryl groups exposed to heat-induced protein unfolding to form disulfide bonds. Meanwhile, Fe(III) provides good conditions for SH oxidation and SH-SS exchange reactions when coated on the surface, which in turn increases the disulfide bonds [51]. The addition of FA and FA/CD, which significantly enhanced the hydrophobic interactions (*p* < 0.05) promoted the aggregation of myosin tails in the gel and facilitated gel formation [52]. However, no significant changes were observed in hydrogen bonding (*p* > 0.05). This suggests that hydrophobic interactions and disulfide bonds are major factors in the thermally induced formation of myofibrillar protein gels with the addition of FA. The same trend was observed in MP reinforced with Fe(III) through surface coating. Upon reinforcement with Fe(III) in an internal cross-linking mode, there was a significant increase in the ionic and hydrogen bonding content of MP (*p* < 0.05), along with a substantial enhancement in hydrophobic interactions. This phenomenon may be attributed to the rise in the net charge quantity on the protein’s surface, a consequence of the addition of Fe(III) and the subsequent chelation of FA with Fe(III). This chelation process serves to shield hydrogen bonds from being disrupted. The implication is that the incorporation of Fe(III) can assist FA in withstanding the oxidation of MP, thereby maintaining the stability of the protein’s structure.

In summary, hydrophobic interactions, disulfide bonds, and hydrogen bonds are the primary forces responsible for the formation of gels by MP. An increase in their concentration facilitates the preservation of the gel’s three-dimensional structure.

#### 3.2.2. Secondary Structure

**Figure 4:**
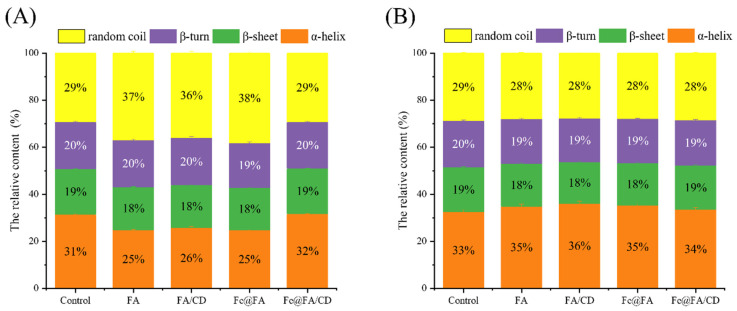
Effects of FA or FA/CD addition and Fe(III) strengthening on secondary structure content of MP gel: (**A**) internal crosslinking; (**B**) surface coating.

As illustrated in Figure 4, the α-helix and irregular coiling configurations constituted the majority of the secondary structure. Figure 4A reveals that the internal cross-linking treatment of MP substantially reduced (*p* < 0.05) the proportion of α-helix and β-sheet structures following the introduction of FA and FA/CD, whereas the proportion of irregular coils experienced a significant increase (*p* < 0.05).

This suggests that thermal induction during gel formation promotes protein unfolding, while different additions and treatments affect the ratio of the secondary structures of myofibrillar proteins. The trend in alterations within the FA group, bolstered by Fe(III), aligned with this pattern, whereas no notable transformation was observed in the FA/CD group (*p* > 0.05). Furthermore, the incorporation of FA/CD led to a substantial rise in the α-helix content of MP. Compared to the FA group (*p* < 0.05), no significant difference was observed in the conformational content of the remaining proteins’ secondary structure (*p* > 0.05). This indicates that the interaction between FA and MP was mitigated by encapsulation with α-CD. The reduced content of α-helix and β-structures suggests that FA may lead to the loosening of protein structures, and promoting cross-linking of proteins [53]. Figure 4B illustrates that the application of surface coating to MP led to an enhancement in the relative abundance of α-helices and a reduction in the relative abundance of β-helixes, β-turns, and irregular coils, following the introduction of FA and FA/CD in comparison to the control. This indicates that the incorporation of FA can prompt the transformation of irregularly curled structures into α-helical conformations [54]. The secondary structure contents of MP remained largely unchanged (*p* > 0.05) following the addition of Fe(III).

It is well-established that protein oxidation results in the degradation of the alpha-helical structure. Consequently, ferulic acids (FA) may play a role in this process by facilitating protein oxidation, which in turn causes the unraveling of the alpha-helix and the subsequent formation of random coil structures. During heating, the alpha-helical structure of myosin unwinds, exposing functional residues, and myofibrillar protein (MP) begins to aggregate, ultimately forming a three-dimensional reticular gel. In this investigation, the incorporation of FA and FA/CD led to the creation of a heat-resistant protein structure within the MP. The underlying cause could be attributed to the covalent attachment of FA to the side chains of the MP, altering the protein’s hydrophilic–hydrophobic equilibrium. This covalent binding of FA to the protein’s side chains likely hinders MP intermolecular interactions by creating a spatial barrier, thereby enhancing the overall protein stability [55].

The impact of polyphenols on the secondary structure of proteins has been extensively documented, yet the findings are inconsistent. The discrepancies observed in these outcomes could be ascribed to variations in protein type, the structure of phenolic compounds, as well as the treatment methods and concentrations employed.

#### 3.2.3. Microstructure

**Figure 5:**
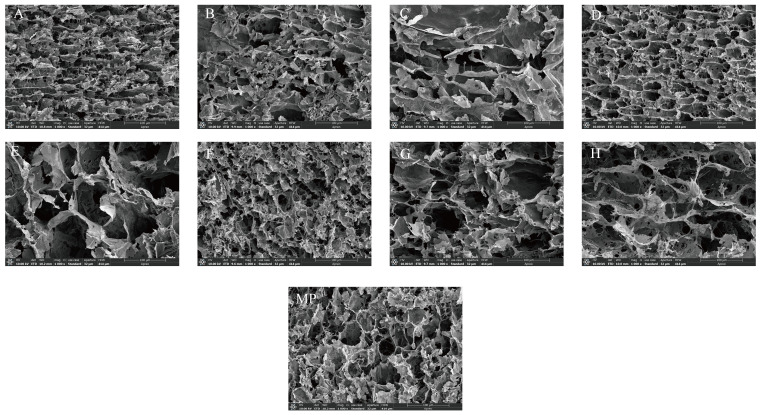
Effects of FA or FA/CD addition and Fe(III) strengthening on microstructure of MP gel: (**A**–**D**) represent FA, FA/CD, Fe@FA, Fe@FA/CD gels treated by surface coating, respectively. (**E**–**H**) represent FA, FA/CD, Fe@FA, Fe@FA/CD gels treated by internal crosslinking, respectively. (**MP**) represents blank gel without any treatment.

To further illustrate the differences in the network structure of MP gels under different treatments, the microstructure of the gels was examined using scanning electron microscopy (SEM), and the results are shown in Figure 5.

The gel network structure of the control group was dense, regular, and featured some smaller pores. This indicates that the protein structure was fully extended and had formed effective cross-linking among the proteins. Upon the addition of FA and FA/CD via surface coating, the pores within the MP gels were diminished, the gel structure became more compact, and the roughness was reduced, resulting in a smoother and more uniform surface. FA enhanced the interaction between protein and water molecules, which was conducive to the internal cross-linking of proteins, forming a tight network structure for the gel. The gel network structure of the MP blank group shows continuity after heat induction. This is because myofibrillar proteins unfold after heating and expose the hidden groups inside. The unfolded myofibrillar proteins interact with each other through covalent and non-covalent bonds to form protein aggregates [56]. However, the gel structure of the MP blank group was looser and had smaller voids. This suggests that there is a problem of poor consistency in the formation of gel structure by thermal induction alone. However, after adding FA and FA/CD by surface coating, the pores in the MP gels were reduced. The gel structure became more compact, the roughness was reduced, and the surface was smoother and more uniform. This is mainly because the addition of FA can induce the unfolding of the protein structure, which exposes the hydrophobic groups to the MP surface and promotes the formation of disulfide bonds between the sulfur-containing amino acid molecules. This promotes cross-linking thus forming a dense and uniform gel structure [57]. Meanwhile, the addition of CD can prevent the excessive modification of MP by FA [50].

Upon the addition of Fe(III), protrusions emerged on the surface of the MP gel, indicating the formation of an Fe-FA complex on the gel’s surface. Furthermore, in the presence of FA, Fe(III) exhibited a plate-like aggregation state. The MP gel network exhibits a sheet-like structure with poor uniformity when observed under a microscope. The Fe(III) -FA coating layer is thin, and the pores within the MP gel are excessively large and irregular, making it challenging to completely cover the macroporous nature of the MP gel. The pores of the MP gel remain visible on the surface, likely due to the reduction in hydrophobic forces between FA and MP by Fe(III). This decrease in binding affinity between FA and MP leads to an uneven distribution of protein aggregates, resulting in a slightly irregular protein network structure. Following internal cross-linking treatment, the surface of the MP gel becomes rough, its network structure loosens, and the distribution becomes uneven, with the emergence of some larger voids. The potential cause for this could be an excessive amount of FA, which hinders the cross-linking process between proteins. The protein network structure experienced damage, resulting in the degradation of the gel network structure. This, in turn, impacted the gel’s water retention capacity and textural properties.

#### 3.2.4. Elemental Analysis

**Figure 6:**
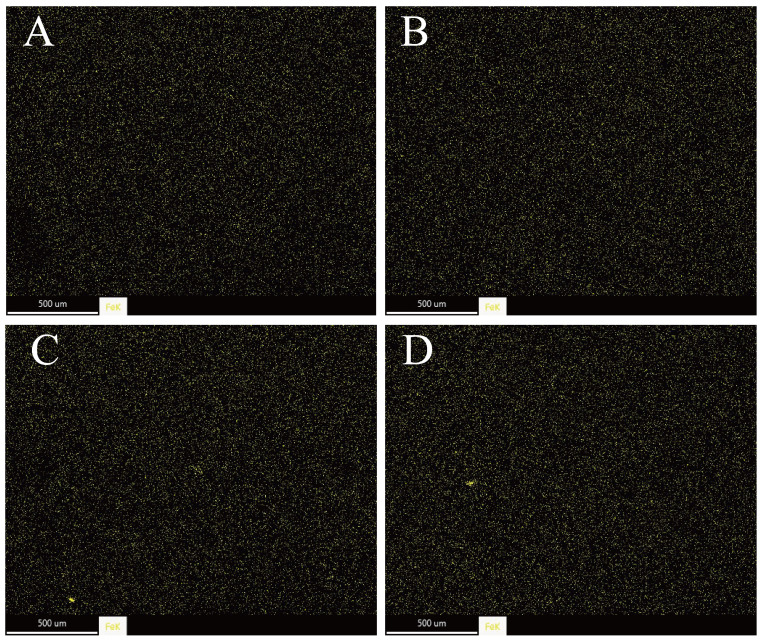
The distribution of Fe elements on the surface of Fe(III) strengthened MP gel in different ways: (**A**,**B**) represent Fe@FA, Fe@FA/CD gels treated by surface coating, respectively. (**C**,**D**) represent Fe@FA, Fe@FA/CD gels treated by internal crosslinking, respectively.

The outcomes of the energy dispersive spectroscopy (EDS) analysis, conducted under 100× magnification, are depicted in Figure 6.

It is evident that within a particular imaging area, Fe(III) ions are extensively dispersed across the entire surface of the MP gel. This observation confirms that the FA-Fe(III) complex has been successfully applied to the MP gel surface.

## 4. Conclusions

FA and Fe(III) improved the MP gel properties to different degrees. The incorporation of FA into the surface-coating process resulted in a substantial enhancement of the hardness, elasticity, chewability, adhesion, and recovery properties of MP gels. Conversely, the internal cross-linking method led to a notable reduction in these same characteristics. The incorporation of FA/CD in either method had minimal impact on the texture of MP, and the introduction of Fe(III) via internal cross-linking further enhanced the textural properties of MP gels. The application of an iron-based surface coating to MP substantially enhanced its water retention capabilities. Furthermore, the incorporation of iron(III) into FA and FA/CD also ameliorated the rheological properties of MP by reducing the gel’s free water content and transforming it into bound, immobile water. Concurrently, hydrophobic interactions, disulfide bonding, and hydrogen bonding are the primary forces responsible for the gelation of MP. By treating MP with internal cross-linking, FA and FA/CD additions reinforced with Fe(III) ions transformed the α-helical structure of the protein to a β-structure or an irregularly coiled structure. However, the treatment of MP by surface coating induced the transformation of the irregularly coiled protein structure to the α-helical structure. Furthermore, the MP gel structure exhibited increased density subsequent to the application of the surface coating, in contrast to the internally cross-linked group. The Fe(III) was evenly dispersed on the internally cross-linked surface, and the MP gel surface exhibited a blocky lamellar structure.

## Figures and Tables

**Table 1:** Effects of FA, FA/CD addition and Fe(III) strengthening (surface coating) on the textural properties of MP gel.

Groups	MP	MP-FA	MP-FA/CD	Fe@MP-FA	Fe@MP-FA/CD
Hardness/g	165.32 ± 8.73 ^a^	144.30 ± 0.16 ^b^	171.65 ± 3.48 ^a^	178.46 ± 7.27 ^a^	145.51 ± 1.34 ^b^
Adhesiveness/g	−0.24 ± 0.02 ^a^	−0.53 ± 0.08 ^b^	−0.60 ± 0.01 ^bc^	−0.81 ± 0.10 ^c^	−0.72 ± 0.07 ^bc^
Chewiness	102.24 ± 4.89 ^a^	82.57 ± 3.24 ^b^	104.87 ± 4.88 ^a^	108.03 ± 3.96 ^a^	81.58 ± 2.36 ^b^
Gumminess	118.40 ± 6.05 ^a^	100.56 ± 1.47 ^b^	125.02 ± 4.42 ^a^	131.77 ± 4.38 ^a^	101.30 ± 3.04 ^b^
Springiness	0.86 ± 0.00 ^a^	0.83 ± 0.01 ^b^	0.84 ± 0.01 ^ab^	0.82 ± 0.00 ^b^	0.81 ± 0.01 ^b^
Cohesiveness	0.72 ± 0.00 ^a^	0.71 ± 0.01 ^a^	0.73 ± 0.01 ^a^	0.74 ± 0.01 ^a^	0.73 ± 0.02 ^a^
Resilience	0.43 ± 0.00 ^a^	0.35 ± 0.00 ^c^	0.40 ± 0.00 ^b^	0.40 ± 0.01 ^b^	0.34 ± 0.00 ^c^

^a–c^ Different letters indicate significant differences between groups (*p* < 0.05).

**Table 2:** Effects of FA, FA/CD addition and Fe(III) strengthening (internal crosslinking) on the textural properties of MP gel.

Groups	MP	MP-FA	MP-FA/CD	Fe@MP-FA	Fe@MP-FA/CD
Hardness/g	113.28 ± 1.17 ^b^	137.91 ± 1.95 ^a^	111.63 ± 2.71 ^b^	129.99 ± 1.13 ^a^	105.75 ± 3.99 ^b^
Adhesiveness/g*s	−0.17 ± 0.04 ^a^	−0.05 ± 0.05 ^a^	−0.06 ± 0.03 ^a^	−0.04 ± 0.02 ^a^	−0.15 ± 0.07 ^a^
Chewiness	58.07 ± 1.46 ^b^	79.63 ± 5.43 ^a^	54.57 ± 3.32 ^b^	73.48 ± 1.90 ^a^	50.97 ± 2.33 ^b^
Gumminess	72.96 ± 1.23 ^b^	95.26 ± 4.61 ^a^	70.26 ± 3.24 ^b^	89.63 ± 1.32 ^a^	66.73 ± 2.65 ^b^
Springiness	0.80 ± 0.01 ^abc^	0.83 ± 0.01 ^a^	0.78 ± 0.01 ^bc^	0.82 ± 0.01 ^ab^	0.76 ± 0.01 ^c^
Cohesiveness	0.64 ± 0.00 ^a^	0.69 ± 0.03 ^a^	0.63 ± 0.01 ^a^	0.69 ± 0.01 ^a^	0.63 ± 0.01 ^a^
Resilience	0.34 ± 0.00 ^c^	0.40 ± 0.02 ^a^	0.36 ± 0.00 ^bc^	0.39 ± 0.00 ^ab^	0.34 ± 0.00 ^c^

^a–c^ Different letters indicate significant differences between groups (*p* < 0.05).

**Table 3:** Effects of FA, FA/CD addition and Fe(III) strengthening (surface coating) on the WHC of MP gel.

Groups	Cooking Loss/%	WHC/%
MP	-	79.16 ± 1.16 ^c^
MP-FA	-	87.08 ± 1.23 ^b^
MP-FA/CD	-	96.23 ± 0.68 ^a^
Fe@MP-FA	-	96.04 ± 2.93 ^a^
Fe@MP-FA/CD	-	94.55 ± 0.65 ^a^

^a–c^ Different letters indicate significant differences between groups (*p* < 0.05).

**Table 4:** Effects of FA, FA/CD addition and Fe(III) strengthening (internal crosslinking) on the WHC of MP gel.

Groups	Cooking Loss/%	WHC/%
MP	4.42 ± 1.14 ^ab^	81.04 ± 0.44 ^a^
MP-FA	5.99 ± 1.28 ^ab^	82.47 ± 2.58 ^a^
MP-FA/CD	7.19 ± 0.96 ^a^	75.51 ± 0.21 ^b^
Fe@MP-FA	3.66 ± 0.37 ^b^	85.34 ± 0.95 ^a^
Fe@MP-FA/CD	6.26 ± 1.17 ^ab^	71.52 ± 0.48 ^b^

^a,b^ Different letters indicate significant differences between groups (*p* < 0.05).

**Table 5:** Effects of FA or FA/CD addition and Fe(III) strengthening (surface coating) on T2 relaxation time of MP gel.

Groups	T_2b_/ms	T_21_/ms	T_22_/ms	T_23_/ms
MP	0.80 ± 0.03 ^d^	14.15 ± 0.27 ^a^	192.49 ± 2.56 ^a^	2416.91 ± 52.94 ^a^
MP-FA	0.87 ± 0.07 ^d^	11.99 ± 0.49 ^ab^	188.17 ± 3.17 ^ab^	2316.35 ± 35.29 ^ab^
MP-FA/CD	1.11 ± 0.04 ^c^	13.85 ± 0.79 ^ab^	186.34 ± 1.70 ^abc^	2198.19 ± 37.77 ^bc^
Fe@MP-FA	1.40 ± 0.05 ^b^	12.62 ± 0.71 ^ab^	180.96 ± 1.41 ^bc^	2097.02 ± 46.79 ^c^
Fe@MP-FA/CD	1.62 ± 0.05 ^a^	11.77 ± 0.54 ^b^	179.64 ± 3.39 ^c^	2080.60 ± 55.46 ^c^

^a–c^ Different letters indicate significant differences between groups (*p* < 0.05).

**Table 6:** Effects of FA or FA/CD addition and Fe(III) strengthening (internal crosslinking) on T2 relaxation time of MP gel.

Groups	T_2b_/ms	T_21_/ms	T_22_/ms	T_23_/ms
MP	0.80 ± 0.03 ^c^	14.15 ± 0.27 ^ab^	192.49 ± 2.56 ^b^	2416.91 ± 52.94 ^ab^
MP-FA	1.21 ± 0.04 ^b^	14.37 ± 0.76 ^ab^	178.20 ± 1.35 ^c^	2320.45 ± 70.74 ^ab^
MP-FA/CD	1.20 ± 0.06 ^b^	14.59 ± 0.83 ^a^	181.09 ± 3.43 ^c^	2303.96 ± 76.96 ^b^
Fe@MP-FA	1.51 ± 0.03 ^a^	12.84 ± 0.48 ^ab^	191.00 ± 1.87 ^b^	2503.83 ± 58.68 ^ab^
Fe@MP-FA/CD	1.46 ± 0.05 ^a^	12.44 ± 0.40 ^b^	179.61 ± 2.14 ^c^	2394.33 ± 82.88 ^ab^

^a–c^ Different letters indicate significant differences between groups (*p* < 0.05).

**Table 7:** Fraction of each relaxation component at FA or FA/CD addition and Fe(III) strengthening (surface coating) of MP gel.

Groups	P_2b_/%	P_21_/%	P_22_/%	P_23_/%
MP	1.22 ± 0.08 ^c^	1.35 ± 0.05 ^b^	87.13 ± 0.43 ^a^	9.58 ± 0.56 ^a^
MP-FA	2.30 ± 0.03 ^b^	2.49 ± 0.11 ^a^	87.95 ± 0.54 ^a^	8.08 ± 0.37 ^a^
MP-FA/CD	2.30 ± 0.05 ^b^	2.48 ± 0.09 ^a^	87.95 ± 0.72 ^a^	7.22 ± 0.60 ^a^
Fe@MP-FA	3.03 ± 0.03 ^a^	2.61 ± 0.03 ^a^	87.36 ± 0.39 ^a^	6.87 ± 0.27 ^a^
Fe@MP-FA/CD	3.17 ± 0.28 ^a^	2.42 ± 0.08 ^a^	86.45 ± 0.48 ^a^	7.58 ± 0.33 ^a^

^a–c^ Different letters indicate significant differences between groups (*p* < 0.05).

**Table 8:** Fraction of each relaxation component at FA or FA/CD addition and Fe(III) strengthening (internal crosslinking) of MP gel.

Groups	P_2b_/%	P_21_/%	P_22_/%	P_23_/%
MP	1.22 ± 0.08 ^c^	1.35 ± 0.05 ^a^	87.13 ± 0.43 ^a^	9.58 ± 0.56 ^a^
MP-FA	1.50 ± 0.10 ^ab^	1.32 ± 0.03 ^a^	87.27 ± 1.23 ^a^	9.25 ± 1.42 ^a^
MP-FA/CD	1.46 ± 0.06 ^abc^	1.37 ± 0.09 ^a^	88.52 ± 0.70 ^a^	8.71 ± 0.74 ^a^
Fe@MP-FA	1.35 ± 0.05 ^bc^	0.98 ± 0.04 ^c^	87.71 ± 0.72 ^a^	8.99 ± 0.74 ^a^
Fe@MP-FA/CD	1.63 ± 0.10 ^a^	1.00 ± 0.02 ^bc^	88.37 ± 0.74 ^a^	9.00 ± 0.73 ^a^

^a–c^ Different letters indicate significant differences between groups (*p* < 0.05).

## Data Availability

The original contributions presented in the study are included in the article, further inquiries can be directed to the corresponding authors.
